# Systematic Review on Parkinson’s Disease Medications, Emphasizing on Three Recently Approved Drugs to Control Parkinson’s Symptoms

**DOI:** 10.3390/ijerph19010364

**Published:** 2021-12-30

**Authors:** Palanisamy Sivanandy, Tan Choo Leey, Tan Chi Xiang, Tan Chi Ling, Sean Ang Wey Han, Samantha Lia Anak Semilan, Phoon Kok Hong

**Affiliations:** 1 Department of Pharmacy Practice, School of Pharmacy, International Medical University, No. 126, Jalan Jalil Perkasa 19, Bukit Jalil, Kuala Lumpur 57000, Malaysia; 2 School of Postgraduate Studies, International Medical University, No. 126, Jalan Jalil Perkasa 19, Bukit Jalil, Kuala Lumpur 57000, Malaysia; 3 Bachelor of Pharmacy (Hons) Programme, School of Pharmacy, International Medical University, No. 126, Jalan Jalil Perkasa 19, Bukit Jalil, Kuala Lumpur 57000, Malaysia; kyajulie1517@gmail.com (T.C.L.); chixiangtan98@gmail.com (T.C.X.); chilingtan0123@gmail.com (T.C.L.); sean9n9@gmail.com (S.A.W.H.); samanthalia96@gmail.com (S.L.A.S.); kokhong98@hotmail.com (P.K.H.)

**Keywords:** brain, neurotransmission, dopamine, monoamine, anticholinergics

## Abstract

Parkinson’s Disease (PD) is a disease that involves neurodegeneration and is characterised by the motor symptoms which include muscle rigidity, tremor, and bradykinesia. Other non-motor symptoms include pain, depression, anxiety, and psychosis. This disease affects up to ten million people worldwide. The pathophysiology behind PD is due to the neurodegeneration of the nigrostriatal pathway. There are many conventional drugs used in the treatment of PD. However, there are limitations associated with conventional drugs. For instance, levodopa is associated with the on-off phenomenon, and it may induce wearing off as time progresses. Therefore, this review aimed to analyze the newly approved drugs by the United States-Food and Drug Administration (US-FDA) from 2016–2019 as the adjuvant therapy for the treatment of PD symptoms in terms of efficacy and safety. The new drugs include safinamide, istradefylline and pimavanserin. From this review, safinamide is considered to be more efficacious and safer as the adjunct therapy to levodopa as compared to istradefylline in controlling the motor symptoms. In Study 016, both safinamide 50 mg (*p* = 0.0138) and 100 mg (*p* = 0.0006) have improved the Unified Parkinson’s Disease Rating Scale (UPDRS) part III score as compared to placebo. Improvement in Clinical Global Impression—Change (CGI-C), Clinical Global Impression—Severity of Illness (CGI-S) and off time were also seen in both groups of patients following the morning levodopa dose. Pimavanserin also showed favorable effects in ameliorating the symptoms of Parkinson’s Disease Psychosis (PDP). A combination of conventional therapy and non-pharmacological treatment is warranted to enhance the well-being of PD patients.

## 1. Introduction

Parkinson’s Disease (PD) is a progressive degenerative neurological disorder commonly presenting with symptoms of muscle rigidity, instability, tremor, bradykinesia (slow in movement), and lack of coordination [1,2,3,4,5,6,7]. PD is the second most frequent age-related neurodegenerative disease which affects up to ten million people worldwide [8]. Non-motor symptoms involve pain, sleep disturbance, psychosis, depression, anxiety, fatigue, and cognitive decline particularly patients aged 40 and above [1,2,4,5,6,9]. All these PD symptoms have significant effects on the quality of life (QOL) of the patients as they feel deteriorating of their own physical functioning. A sense of being deprived of one’s self-worth, feeling isolated in society, speech problems are always the problems faced by a PD patient daily [4,10]. PD has a few complications, one of them is Parkinson’s Disease Psychosis (PDP) where the signs and symptoms are the same as psychosis except that the sensorium of PDP patients is clear [6]. PDP impacts QOL and increases the mortality and morbidity of patients [5,6,9]. It occurs in 43–60% of PD patients [9]. Other than affecting patients’ QOL as a non-motor symptom, it also increases caregiver stress and burden as well as increases the chances of nursing home placement for the patients [5,6]. PDP management remains a challenge for the healthcare community because dopaminergic therapies for PD motor symptoms treatment will exacerbate PDP [9]. Besides that, antipsychotic usage in PD treatment will also increase the mortality and morbidity rate as compared to placebo [6]. Other than PDP, PD patients will also experience pain which deteriorates their QOL [1,4]. As comorbidity of PD, pain may overshadow the motor symptoms of PD [4]. To find new ways of controlling PD symptoms, novel drugs such as safinamide, istradefylline, and pimavanserin were developed and approved from 2016 to 2019. This review is conducted to examine the efficacy and safety of safinamide, istradefylline, and pimavanserin as adjuvant therapy in controlling Parkinsonism symptoms.

In America, approximately 60,000 citizens are diagnosed with PD each year, with a higher rate in males (1.5 times more prominent) compared with females [11]. The prevalence of PD is constantly rising with age, influencing 1% of the population above 60 years old and up to 4% of the population above 80 years old [4,12]. The incidence, or the rate of newly diagnosed PD, is expected to double by 2030 in industrialized countries, including the United States [13]. According to a systematic analysis, PD prevalence in 2016 in Malaysia is 514 cases (95% UI 386–672); Indonesia is 3490 cases (95% UI 2682–4487); India is 17,539 cases (95% UI 13317–22,637); China is 40,012 cases (95% UI 31,132–51,074) [14]. Because of the highest prevalence rate, the number of patients with Parkinson’s disease in this region is expected to more than fivefold by 2040, from an estimated 20,000 to 120,000 [15].

Familial Parkinson disease (FPD) is an inherited form of PD caused by a mutation in the alpha-synuclein gene (SNCA) on chromosome 4q2, whereas the sporadic form of PD (SPD) can affect anyone and is caused by environmental toxins that lead to alpha-synuclein accumulation [16]. In most cases, the cause of PD is idiopathic [12]. Researchers believe that it is caused by a combination of factors, with both genetic and environmental causes playing an important role. However, not everyone with a genetic mutation will develop PD. Similarly, not everyone exposed to pesticides will be diagnosed [17]. This makes the study of the etiology of PD challenging. Nevertheless, aging is the greatest risk factor for the development of PD [18]. Antipsychotics, antiepileptic drugs, and calcium channel blockers (CCBs) have been found to induce PD, which also known as drug-induced parkinsonism [19]. Several small strokes followed by ischemic changes in the brain will cause Parkinson’s-like symptoms, which is also known as arteriosclerotic parkinsonism [20].

PD is a condition that is characterized by various motor symptoms and is due to the neurodegeneration of the nigrostriatal pathway [21]. There are several factors that will initiate neurodegeneration, which includes ageing, ethnicity, pesticides, family history, genetics, radiation, and trauma or infection. The dopamine level drops due to the degeneration of the substantia nigra neurons which results in the disruption in the thalamus and motor cortex connection [22]. The reduced level of dopamine initiates changes in the density and sensitivity of the dopamine receptors due to compensatory mechanism [23]. The dopamine D1 and dopamine D2 receptors are activated by the dopaminergic neurons in the dorsal striatum which originates from the substantia nigra and terminates in the caudate and putamen [24]. When dopamine level drops, it will result in the relative over-activity in the indirect pathway due to disinhibition of the substantia nigra. The striatum extends to the external globus pallidus in the indirect pathway, using GABA as a neurotransmitter. Then, the external globus pallidus extends to the substantia nigra which is responsible for providing excitatory input by utilizing glutamate as the neurotransmitter. Reduced inhibition that exerts on the direct pathway causes an additional disinhibition of the output nuclei which is the internal globus pallidus and substantia nigra. The internal globus pallidus output nuclei inhibit the thalamus at a higher intensity and lesser excitatory input to the motor cortex [25]. This causes the main pallidal-thalamic outflow pathway to exhibit too much inhibitory signal to the thalamus which in turn results in suppression of the thalamo-cortical-spinal pathway. Thus, it leads to parkinsonian signs such as bradykinesia. However, the involvement of basal ganglia in the pathophysiology of PD is not clear [26].

The reduced level of dopamine in the brain can be restored by a compensatory mechanism to mask the deleterious effect of the depletion of dopamine levels. The brain can increase dopamine levels by elevating the production of dopamine [27]. Another compensatory mechanism is the reduction of the dopamine transporter, which results in less dopamine neurotransmitter reuptake and restoring the dopamine level [28].

**Figure 1:**
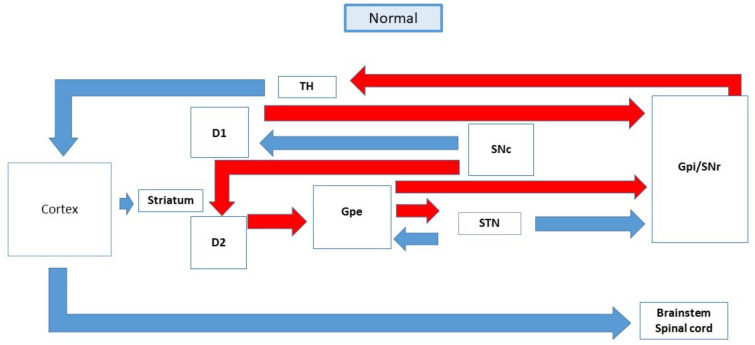
The schematic diagram of the basal ganglia and their connections in normal patient.

**Figure 2:**
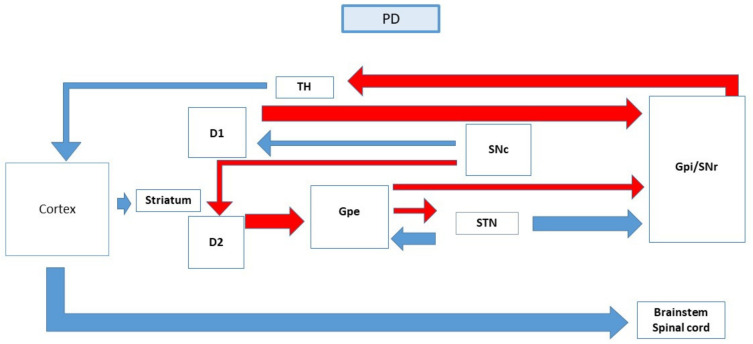
The schematic diagram of the basal ganglia and their connections in Parkinson’s Disease patient.

The mitochondria dysfunction and protein aggregation also play a vital role in the development of PD. When protofibrils are translocated to the membranes, Abeta peptide and alpha-synuclein may interact to cause mitochondrial and plasma membrane damage. The accumulation of Abeta and alpha-synuclein oligomers in the mitochondrial membrane may result in the release of cytochrome C, triggering the apoptosis cascade. Conversely, the oxidative stress and mitochondrial dysfunction associated with Alzheimer’s and Parkinson’s disease may increase membrane permeability and cytochrome C release, promoting Abeta and alpha-synuclein oligomerization and neurodegeneration [29] [Figure 1 and Figure 2].

## 2. Methodology

A Systematic literature search on Parkinson disease, Parkinson, Parkinsonism, safety, and efficacy of Parkinson drugs was conducted in Science direct, PubMed, and Google Scholar using MeSH terms such as Parkinson disease, Parkinson, Parkinsonism, Outbreak, Epidemic, Safety, and Efficacy. All published articles from 2016 to 2019 were screened. In addition to that, Centers for Disease Control & Prevention (CDC), World Health organization (WHO), US Food & Drug Administration, and UpToDate websites also searched for the latest reports, guidelines, approved drugs, etc. The cross references pertaining to the important research and review articles were also considered. Articles which are relevant to the aim of the current review were selected. The drugs included in our studies are those that were approved by US-FDA from the year 2016 to 2019, as according to Centre Watch. There were 1210 articles on the topic of the review screened and 47 articles selected for the final review. Only the RCT and studies on human subjects were considered, animal studies were excluded. The authors independently extracted relevant information from randomized control trials (RCT) that fulfilled our inclusion criteria, and any disagreements were resolved through consensus. The extracted data included the trial phase, region, subject conditions, and outcome measures. The data of each drug was compiled and summarized comprehensively into paragraphs. The following search strategy was used in this review for the selection of the article.

(“parkinsonian disorders” [MeSH Terms] or (“parkinsonian” [All Fields] and “disorders” [All Fields]) or “parkinsonian disorders” [All Fields] or “parkinsonism” [All Fields]) and (“dopamine” [MeSH Terms] or “dopamine” [All Fields]) and (“istradefylline” [Supplementary Concept] OR “istradefylline” [All Fields]) or (“safinamide” [Supplementary Concept] or „safinamide” [All Fields]) or (“pimavanserin” [Supplementary Concept] or “pimavanserin” [All Fields]) and (“loattrfree full text” [sb] and “16 January 2015” [PDat]: “14 January 2020” [PDat] and “humans” [MeSH Terms]).

This systematic review is registered with the International Prospective Register of Systematic Reviews (PROSPERO, registration number: CRD42021282847).

## 3. Management of Parkinson’s Disease and Its Associated Psychosis

There are conventional drugs used in treating PD such as levodopa, dopamine agonists, anticholinergics, catechol-O-methyltransferase (COMT) inhibitors, monoamine oxidase-B (MAO-B) inhibitors, and amantadine. Other drugs used in treating PD related psychosis include antipsychotics. During this review period, three drugs were newly approved by the FDA for the treatment of PD symptoms and PDP, which were safinamide, istradefylline, and pimavanserin. Their mechanism of action, efficacy, and safety will be discussed below.

### 3.1. Mechanism of Action

The mechainsm of action of drugs that are commonly used for the treatment of PD and its symptoms are describe below.

#### 3.1.1. Levodopa

The mode of action of levodopa involves absorption from the gastrointestinal tract, crossing through the blood-brain barrier (BBB), uptake by neurons, enzymatic action of the aromatic amino acid decarboxylase to be converted into dopamine and the synaptic release of dopamine [30]. The disruption of the nigrostriatal pathway reduces dopamine levels and produces the symptoms of PD [31]. Hence, the dopamine from exogenous levodopa will activate the central dopamine receptors, thus improving the symptoms of PD. Since aromatic-L-amino-acid decarboxylase (AADC) and COMT are responsible for the metabolism of levodopa peripherally, it is usually in combination with AADC inhibitors such as carbidopa and benserazide or COMT inhibitors such as entacapone and tolcapone. Levodopa has to be administered multiple times daily since it has a short half-life of about 36–96 min which will cause the fluctuation in plasma levels [30]. Treatment with Levodopa alleviates bradykinesia and other typical motor manifestations of PD. Long-term Levodopa treatment, on the other hand, is associated with complications such as motor fluctuations and dyskinesia, which severely impair quality of life. The combination of levodopa and carbidopa is most widely used to treat PD and Parkinson-like symptoms that may develop after encephalitis (brain swelling), or nervous system injury caused by carbon monoxide or manganese poisoning [32].

#### 3.1.2. Dopamine Agonists

Dopamine agonists can be categorized into two classes, which are ergot and non-ergot dopamine agonists. They have antiparkinsonian activity due to the direct-acting effect on dopamine receptors which mimic the neurotransmitter. Bromocriptine, cabergoline, pergolide, and lisuride are examples of ergot dopamine agonists whereas non-ergot dopamine agonists include ropinirole and pramipexole. Ergot dopamine agonists act primarily on the D2-like dopamine receptors including D2, D3, and D4 [33]. On the other hand, non-ergot dopamine agonist ropinirole is a potent and selective agonist of the D2 dopamine receptors while pramipexole has a higher affinity towards D3 receptors [33,34].

#### 3.1.3. Anticholinergics

In PD patients, it has been theorized that a lesion is formed in the nigra striatum. This results in the reduction of intranigral dopamine concentrations. Imbalances of the dopaminergic and cholinergic neurological pathway lead to more cholinergic firing. The stimulation causes dyskinesia and tremors. Thus, the mechanism of anticholinergics is to block the cholinergic receptors from the activation of acetylcholine. They act to counteract the imbalance of neurotransmitters in the nigra striatal pathway [35]. Specifically, M4 receptor is targeted for the block by anticholinergics [36]. This eventually will reduce the tremor and dyskinesia conditions of the patient.

#### 3.1.4. COMT Inhibitors

A COMT inhibitor acts by breaking down catecholamines such as dopamine and norepinephrine by inhibiting the enzyme COMT. The enzyme COMT can be found in peripheral and central circulation and the aim is to prevent the breakdown of levodopa while travelling to the brain region and crossing through the BBB. It works in combination with levodopa to prevent methylation of levodopa to 3-O-methyldopa in peripheral circulation, thus improving the bioavailability of levodopa [37]. Besides, low doses of levodopa in combination with COMT inhibitors may prevent dyskinesia.

#### 3.1.5. MAO-B Inhibitors

MAO-B inhibitors (MAO-BIs) are the antiparkinsonian drugs that have the mechanism of action of preventing monoamine oxidase-B (MAO-B) from catalyzing dopamine metabolism, hence prolonging dopamine action in basal ganglia. It is considered as an adjuvant for PD, and it is usually used with L-Dopa for PD therapy. Besides PD, MAO-BIs are also the adjuvant for treating Alzheimer’s disease. MAO-BIs exhibit neuroprotection which is the protective effect of neuronal structure and function. Other than that, they also exhibit antioxidant effects and can prolong neuronal death caused by apoptosis as well as protect functions of mitochondria [38]. Current MAO-BI consists of selegiline and rasagiline. Both are selective and irreversible MAO-BIs [39].

#### 3.1.6. Amantadine

The mechanism of amantadine in the brain is not well understood. Generally, amantadine works by inhibiting the N-methyl-D-aspartate (NMDA)-glutamate receptor and cholinergic muscarinic receptors, thereby increasing dopamine release, and blocking dopamine reuptake [40]. It reduces dyskinesia in PD patients receiving levodopa, as well as extrapyramidal side effects of medications. Multiple studies showed that NMDA-blocking is the most important mechanism to explain its antidyskinetic effect [41].

#### 3.1.7. Antipsychotics Used for Treating PDP

##### Clozapine

Clozapine is an FDA-approved tricyclic dibenzodiazepine antipsychotic drug commonly used in schizophrenia patients [42,43,44]. It is classified as an ‘atypical’ antipsychotic due to the selectivity of binding towards the dopamine receptors that differ from typical antipsychotic drugs. In this review, clozapine is focused as an off-label used in psychosis in PD patients. Psychosis happens when there is excessive dopamine level while clozapine able to antagonist the dopamine receptor to control the dopamine level. It has a high affinity towards dopamine D4 receptors while also targets D1, D2, D3, and D5 [34,43,44]. In other words, clozapine is more likely to act on limbic rather than striatal dopamine receptors thus reducing the psychosis symptoms in the parkinsonian patients [43,44]. In addition, clozapine is found to have an antagonistic effect on adrenergic (alpha-1), cholinergic (muscarinic M1, M2, M3, and M5), histaminergic, and serotonergic receptors [34,43,44]. Evidence has also shown that serotonin 2A receptors neurotransmission abnormalities are associated with psychosis in PD patients [45]. Clozapine has great efficacy in managing psychosis in PD but is underused due to its potential adverse events.

##### Olanzapine

Olanzapine is a second-generation antipsychotic that produces its effect on the dopamine as well as serotonin receptors. It works primarily on the mesolimbic pathway dopamine 2 receptor as a blocker [11]. It blocks the dopamine neurotransmitter from exerting the effects on the postsynaptic receptor. Olanzapine binds to the receptor loosely and so enables the normal amount of dopamine to carry out neurotransmission [46]. The effects of olanzapine on the dopamine 2 receptor led to the reduction in positive symptoms in the patient, which includes hallucination, delusion, and disorganized speech. As for the serotonin 5-HT2A receptors, the olanzapine works as an antagonist as well. Since serotonin 5-HT2A receptors are located in the frontal cortex, this results in reduced negative symptoms which include anhedonia, flat affect, alogia, and poor attention [47].

##### Quetiapine

Quetiapine, a dibenzothiazepine atypical antipsychotic, has a similar action to clozapine whereby it inhibits D2 receptors and serotonin 5-HT2A receptors. It also binds to serotonin 5-HT1A, D1, H1, alpha 1, and alpha 2 receptors [48]. Nowadays, quetiapine is the most widely used antipsychotic in the treatment of PDP as monitoring for blood dyscrasias is not required and it shows the minor effect on motor symptoms [49].

##### Risperidone

Risperidone is a benzisoxazole atypical antipsychotic which has high antagonistic activity on the 5-HT2A and D2 receptors [50]. Thus, it can cause a harmful effect on dopamine replacement therapy and can aggravate motor symptoms [49]. Unlike clozapine, risperidone does not cause seizures, and hematologic and antimuscarinic side effects [51]. In people with schizophrenia, risperidone does not cause more extrapyramidal symptoms at doses less than 6 mg/day compared to placebo as serotonin antagonism will be predominant at low doses. Furthermore, low doses of risperidone cause progressive occupancy of dopamine D2 receptor in comparison to typical neuroleptics, modulation of the dopamine system by serotonin 5-HT2 antagonism, and selective mesolimbic blockage instead of striatal dopamine receptors [51].

##### Ziprasidone

Ziprasidone is a second-generation atypical antipsychotic with the chemical structure of benzylisothiazolylpiperazine. It has inhibitory effects on D2, 5-HT2A, and 5-HT1D receptors as well as agonistic effects to 5-HT1A receptors. The inhibitory effect for the reuptake of norepinephrine and serotonin is moderate [48]. Among PDP medications, it is deemed to be efficacious and safe due to its profile of efficacy and safety [52].

#### 3.1.8. Safinamide

Safinamide is an FDA newly approved drug used in treating PD. It is a derivative of benzylamino which has various modes of action [2,53]. The main mode of action of safinamide is that it inhibits MAO-B selectively and reversibly [2,7,11,53,54]. Moreover, safinamide has the non-dopaminergic mechanism of action which includes the state-dependent block of voltage-gated sodium channels in the inactivated state. Furthermore, safinamide also has antiglutamatergic activity. These actions may be responsible for its pain mitigating effects [2,7,11,53,54]. Safinamide also prevents the formation of free radicals through the inhibition of MAO-B [53].

#### 3.1.9. Istradefylline

Istradefylline is known as the selective adenosine A2A receptor antagonist [3]. Adenosine A2A receptors are demonstrated to suppress the activity of GP projection by suppressing GABA which is transmitted and released in the striatum. This will lead to the enhancement of GABA in the GP [55]. Therefore, when istradefylline blocks A2A receptors, it can reduce outrageous excitability of the indirect output pathway, thereby minimizing the occurrence of the motor symptoms in PD patients [56]. In addition, istradefylline is considered as nondopaminergic due to the lack of effects on dopamine receptors and dopamine-metabolizing enzymes [57]. It does not have the inhibitory activity toward enzymes such as COMT, MAO-A, and MAO-B which metabolize dopamine or levodopa. It also has a low affinity for receptors such as dopamine (D1, D2), serotonin (5-HT1A, 5-HT2, 5-HT3), and noradrenaline receptors [50].

#### 3.1.10. Pimavanserin

Pimavanserin is known as the selective antagonist or inverse agonist of 5-hydroxytryptamine (HT)2A receptor [1,49,58,59,60]. This is because of its ability to reduce 5-HT2 receptor activity without acting on other receptors. Thus, it does not induce a pharmacological reaction to agonists on other receptors [49]. Pimavanserin has a 40 folds higher affinity towards 5-HT2A receptors compared to 5-HT2C receptors [60]. However, it has a low affinity towards dopaminergic, muscarinic, histaminergic, or adrenergic receptors and has a low ability in blocking D2 receptors [1,49,59]. Therefore, the deleterious effect of pimavanserin on dopamine replacement therapy will not be the same as atypical psychotic drugs and it does not worsen motor symptoms [49]. Thus, pimavanserin is the first medication licensed in the United States for the treatment of Parkinson’s disease psychosis (PDP)-related hallucinations and delusions, eventually making pimavanserin the first agent approved by FDA in 2016 for treatment of PDP [1,5].

### 3.2. Efficacy

The efficacy of drugs that are commonly used for the treatment of PD and its symptoms are describe below. 

#### 3.2.1. Levodopa

Levodopa is the most effective drug for the treatment of the symptoms due to the dopaminergic deficit in PD patients. It is always used together with a decarboxylase inhibitor in a fixed combination [57]. However, it does not affect disease progression. The effects of levodopa are dose-dependent. Levodopa is recommended in all stages of PD including early monotherapy, patients with or without motor fluctuations with the presence or absence of dyskinesia, patients with non-motor symptoms and motor complications [61].

Levodopa efficacy and side effect profiles have shown that it is superior to other PD medications such as MAO-B inhibitors, anticholinergics, dopamine agonists, and amantadine [61,62]. However, it is common that motor fluctuations and dyskinesia wear off after the initial period with a continuous clinical response which is also known as “honeymoon period” [61]. A placebo-controlled study of levodopa has demonstrated that a daily dose of 600 mg levodopa induced wearing off in 30% patients and dyskinesia in 17% of patients after 40 weeks of treatment [61,63]. The common pharmacological interventions to reduce the off time include dose adjustment of levodopa, changing formulation of levodopa or the addition of dopamine agonists such as ropinirole or pramipexole. Dopamine agonists can delay the time for the onset of motor complications if they are given before levodopa is started for treatment [61]. Other approaches include prolonging the duration of the effect of levodopa by blocking the enzymes responsible for the degradation of levodopa or dopamine such as adding the MAO-B inhibitors or COMT inhibitors [64].

#### 3.2.2. Dopamine Agonists

Dopamine agonists in combination with levodopa are recommended to be used as single-drug therapy in early PD. They can also be used as the adjuvant to levodopa in treating PD patients with or without motor fluctuations. In terms of the incidence of dyskinesia, dopamine agonists are not more effective than the initial single-drug therapy with levodopa [61]. As compared to levodopa monotherapy, there is a lower incidence of dyskinesia in the following three to five years if dopamine agonists are used for the initial treatment. However, the benefit has not been shown in long term yet [61,62,65,66,67].

#### 3.2.3. Anticholinergics

Benztropine and trihexyphenidyl are anticholinergics that are efficacious for the symptomatic control of parkinsonism [68]. However, the clinical data is insufficient to establish a long-term efficacy of anticholinergic treatment. Anticholinergics are not suitable for PD prevention as well as the control of motor complications. The anticholinergics serve as a single-drug therapy or as an adjuvant to the other antiparkinsonian medications. As compared to placebo, anticholinergics are more effective in enhancing the motor performance in PD [35].

#### 3.2.4. COMT Inhibitors

Entacapone and tolcapone are two COMT inhibitors that are used in the management of PD. Tolcapone is more potent as compared to entacapone but is associated with hepatotoxicity [69]. There were three cases of fulminant hepatitis with the use of tolcapone whereby many countries have withdrawn it from the market. However, tolcapone was reintroduced, but with requirements for monitoring of serum enzymes [70]. A third COMT inhibitor, opicapone, is available in Europe but has not yet been approved by the FDA. Opicapone’s efficacy does not vary much from entacapone, and it needs additional studies to assess its cost-effectiveness [69,71,72]. Tolcapone therapy resulted in a noticeable decrease in the baseline dose of pooled L-dopa relative to entacapone and opicapone (*p* > 0.001). The reduction in pooled dose was as follows: 155 (±720) mg for tolcapone, 48 (±37) mg for entacapone, and 22 (±6) mg for opicapone [69].

#### 3.2.5. MAO-B Inhibitors

DATATOP trial was conducted to study the effect of selegiline as compared to tocopherol, their combination, and placebo based on UPDRS motor scores and the activities of daily living (ADL) score. Based on the trial, it was found that selegiline was attributed to the delay of levodopa administering for nine months. The extension of the study has also discovered that patients administered with selegiline were less prone to on-off motor fluctuations, freezing of gait in comparison with placebo. Slower decrease of UPDRS scores in the active arm was also attributed to selegiline intake. Newer studies have also found out that MAO-BI therapy was able to reduce dyskinesia which the reduction effect was most prominent after two years of administration. In accordance with TEMPO and ADAGIO studies, early rasagiline therapy improved motor function significantly in comparison to the delayed start of rasagiline therapy. Rasagiline can also improve PD motor symptoms such as bradykinesia, tremor, postural instability, and gait. Besides, it can also be the adjunct therapy to dopamine agonists or COMT inhibitors which may improve the motor function and reduce off time [39].

#### 3.2.6. Amantadine

Amantadine was the only drug which showed a significant effect in reducing dyskinesia in PD patients [73]. A meta-analysis from two randomized controlled trials showed that amantadine was effective in reducing levodopa-induced dyskinesia in PD patients. It also decreased the Unified Dyskinesia Rating Scale (UDysRS) total score and on time without troublesome dyskinesia [74]. This can be seen in the study of Oertel et al., who found that amantadine caused more than a two-fold reduction in UDysRS, and at the same time reduced the off time [75].

#### 3.2.7. Antipsychotics Used for Treating PDP

##### Clozapine

In 60 patients with an underlying cause of PD and drug-induced psychosis of at least four weeks duration, a randomized controlled trial was performed with low doses of clozapine (6.25 to 50 mg per day). The results showed that the patients in the clozapine group improved significantly as compared to the placebo group. In the study, the Clinical Global Impression Scale (CGI) was used to measure the psychosis intensity and The Brief Psychiatric Rating Scale (BPRS) rates positive and negative symptoms to assess the intensity of schizophrenia. The findings showed mean (±SE) scores on the CGI improved for the clozapine group as compared to placebo. Hallucinations also improved for the clozapine group in comparison with the placebo (*p* = 0.002) on the BPRS [76]. Another randomized controlled trial in 60 patients with PD revealed that clozapine at a dose lower than 50 mg daily improved psychosis in PD without worsening patients’ motor function [77]. Similar to the previous study, CGI and Positive and Negative Syndrome Scale (PANSS) were chosen as a reference scale for the semi-quantitative evaluation of psychiatric symptoms in schizophrenia in which positive syndrome such as hallucinations or delusions were considered as outcome variables of specific interest in dopaminometic induced psychosis [77].

However, in both studies, patients were withdrawn during the study due to reported adverse effects of neutropenia [42,48,76,77,78,79,80,81]. Although clozapine has shown efficacy in both studies, due to its safety profile and frequent monitoring of blood samples required, other antipsychotic drugs are preferred over clozapine [78,79]. However, in a meta-analysis performed by Helge Frieling et al. in determining the most efficient neuroleptic medication has found out that clozapine can be explicitly recommended for PD psychosis care [48,80,82,83].

##### Olanzapine

In open-label studies, it was shown that olanzapine was effective in parkinsonian patients with psychosis [79]. However, placebo-controlled and comparative trials suggested a worsening of motor symptoms without causing improvement in hallucination [84,85]. In a placebo-controlled, double-blind study, olanzapine treated patients were shown to have improvement on UPDRS part II and structured interview for hallucinations, but they did not reach statistical significance. There were also no significant differences in neuropsychological test battery for olanzapine as compared with placebo [84]. Hence, olanzapine was not shown to have significant efficacy in PD patients with psychosis for improving hallucinations and the drug was associated with the potential of motor function worsening [84].

##### Quetiapine

The earliest study was a randomized 12-week open-label, blinded rater trial of 45 patients with PDP which demonstrated that quetiapine was equally effective as clozapine in reducing psychosis in PD patients, as measured by BPRS and CGI [48,80]. However, another 3-month, double-blind trial of 58 PDP patients showed no significant changes in psychotic outcomes with quetiapine [48]. Although the earlier findings showed the efficacy of quetiapine in treating PDP, recent studies have failed to duplicate the findings [79]. Even though the evidence for the efficacy of quetiapine is insufficient, low-dose quetiapine is still preferred over clozapine as clozapine requires monitoring of clozapine-induced agranulocytosis [80].

##### Risperidone

Risperidone is classified as an atypical antipsychotic medication that is normally used to treat PDP. A very low dose of risperidone is usually well-tolerated by PDP patients [42,49,51]. A study noted that there was no change of motor functioning in patients who were taking risperidone dosage less than 6 mg/kg [42,51]. Similarly, a study shows that 2 groups of subjects who were given 1 mg/day risperidone did not suffer from psychotic symptoms. Nevertheless, risperidone caused the worsening of motor symptoms in both groups where one of the groups was treated with risperidone monotherapy while the other received quetiapine treatment at first before changing to risperidone [49]. This showed that the result from the use of risperidone in treating PDP is varied. In a randomized trial and double-blind study, the efficacy of risperidone and clozapine in decreasing the symptoms of psychosis was similar while risperidone can aggravate extrapyramidal symptoms [51]. In addition, all 172 PD subjects in a retrospective study who were receiving an average daily dose of 1.5 mg risperidone had experienced aggravation of extrapyramidal symptoms [86].

##### Ziprasidone

Based on Pintor et al., 2012, ziprasidone was compared with clozapine in a 4-week randomized trial. The 14 participating PD patients were given with single-blindly clozapine or ziprasidone from which it was used to compare the efficacy of both drugs on psychotic symptoms. The study found out that the reduction of psychotic symptoms assessed with Scale for the Assessment of Positive Symptoms (SAPS) and BPRS, were more intense in the ziprasidone group but with no statistical difference as compared with the clozapine group [52].

Besides that, a 3-months study of ziprasidone was conducted by Gómez-Esteban et al., 2005, in which 10 PD patients with psychotic symptoms that were uncontrollable by adjusting dopaminergic drugs, were given ziprasidone 20–40 mg. Based on the Neuropsychiatric Inventory (NPI), there was greater improvement in psychotic symptoms of the last visit with a higher drug dose as compared to the improvement of the first visit with a lower dose [87]. It can be said that a higher dose with a longer duration of intake led to a better outcome as compared to a lower drug dose at the beginning of therapy.

#### 3.2.8. Safinamide

Safinamide is effective as an adjuvant to levodopa in mid-to-late stage PD patients who have fluctuations in motor function. In the 24-week, double-blind, placebo-controlled SETTLE study which enrolled patients who were treated with optimized doses of levodopa and dopamine agonists, COMT inhibitor, anticholinergics, and/or amantadine, safinamide (50 mg or 100 mg) increased the on time without dyskinesia that was troublesome as compared to placebo [11,88]. Both doses were considered to be effective when they were added to levodopa alone or with other medications with antiparkinsonian effect [11]. In patients with dyskinesia, the dose of 100 mg was found to be better in controlling motor fluctuations [89]. Study 016, A Phase III trial has demonstrated that there was an improvement in UPDRS part III as compared to placebo in both 50 mg and 100 mg safinamide group [90]. Following the morning dose of levodopa, there was also an improvement in Clinical Global Impression—Change (CGI-C), Clinical Global Impression—Severity of Illness (CGI-S) and off time in both groups of patients as compared to placebo [90]. Significant improvements were also reported in the off time, on time without dyskinesia, CGI-S, CGI-C (safinamide 50 mg), UPDRS part II, part III, and part IV total scores in the extension study of Study 016 which was Study 018 [91]. Both Study 016 and SETTLE study have shown that safinamide was safe and effective as an adjuvant to levodopa or dopaminergic therapies. Safinamide was found to improve the symptoms of bradykinesia, tremor, rigidity, and gait [92].

A randomized, double-blind, placebo-controlled trial which was carried out in idiopathic PD patients has found out that the UPDRS part III improved as compared to baseline [53,93]. In another open, single-center, pilot trial, high doses of safinamide (100, 150, and 200 mg daily) were also found to have significant improvement in motor function as evaluated by UPDRS part III [94]. The 015 study has also shown that there was a remarkable improvement in UPDRS part II, UPDRS part III, and CGI-C total score in the safinamide 100 mg group [95]. Study 017, which was a 12-month extension study of the 015 study has demonstrated that there was a lower rate of intervention in the safinamide 100 mg group compared with dopamine agonists monotherapy. The study also suggested that safinamide is effective as an adjuvant to dopamine agonists as shown by the improvement in UPDRS part II and part III [96]. The MOTION study, on the other hand, showed an improvement in UPDRS part III and Parkinson’s disease questionnaire (PDQ)-39 in the safinamide 100 mg group of patients who were on single-drug therapy with a dopamine agonist as compared to placebo [97].

The non-dopaminergic mode of action of safinamide may contribute to its pain mitigating effects as shown by studies [2]. It was found that safinamide was effective as an adjunct in alleviating pain in PD patients and was more efficacious as compared to cannabinoids and opioids [2]. A post hoc analysis of the data from the Study 016 and Study 018 found that safinamide has improved the Parkinson’s Disease Quality of Life Questionnaire (PDQ-39) items. Moreover, safinamide reduced the number of concomitant pain treatments [7,54]. Hence, safinamide was efficacious in alleviating pain symptoms in PD patients.

#### 3.2.9. Istradefylline

In a double-blinded and randomized placebo-controlled study, the efficacy of istradefylline in improving motor function in PD patients was demonstrated. In this study, the subjects with motor complications and on levodopa therapy were randomly assigned to receive istradefylline 20 mg/day, 40 mg/day, or placebo for twelve weeks. The result showed that a daily dose of 40 mg istradefylline caused a remarkable reduction in the UPDRS Part III as compared to istradefylline 20 mg/day [57]. This study also claimed that the group who received 20 mg/day and 40 mg/day showed a statistically significant improvement in CGI-I as compared to placebo. Thus, in 2013, the use of istradefylline in treating wearing off in Japanese PD patients was approved under the co-administration of levodopa. A study also showed that 20 mg/day of istradefylline reduced off time by 1.31, compared to istradefylline 40 mg/day and placebo group which reduced off time 58 h and 0.66 h respectively [6]. Similarly, istradefylline was found in clinical studies to be effective in reducing motor fluctuation without increasing troublesome dyskinesia [50].

#### 3.2.10. Pimavanserin

Studies have shown that pimavanserin demonstrated efficacy in treating PDP The recommended dose of pimavanserin is 34 mg taken orally once daily [1,9,58,61,78,98,99]. Meanwhile, for patients who are taking strong CYP3A4 inhibitors such as ketoconazole, the recommended dose is 10 mg once daily [98]. The efficacy of pimavanserin in treating PDP related delusions and hallucination was first noted in two placebo-controlled clinical trials in PDP [61]. Similarly, 34 mg of pimavanserin was shown to be efficacious for treating delusions and hallucinations in PDP patients in a pivotal trial. An improvement of 5.79 points was observed in the group with pimavanserin while the placebo group only had an improvement of 2.73 in the Scale for the SAPS-PD score. It was also demonstrated that pimavanserin was well-tolerated with negative impacts on patients’ motor functions [58].

In the aspect of concomitant use, one study demonstrated that most of the concomitant use of quetiapine with pimavanserin was well-tolerated among the patients. However, there were 63% of the patients for whom the dosage of quetiapine was increased not more than 34 mg daily at some stage after starting pimavanserin due to the increment of psychosis with disease progression. This study also reported that the lowest possible dose of quetiapine can be added afterward in treating resistant, residual, or newly emerging psychotic symptoms [1].

### 3.3. Safety

The safety of drugs that are commonly used for the treatment of PD and its symptoms are describe below. 

#### 3.3.1. Levodopa

The adverse events associated with levodopa therapy were gastrointestinal side effects, psychological and sleep disturbance as reported in one of the open-label, pragmatic randomized trials [62]. In another randomized, placebo-controlled, double-blind trial, the common adverse event which was due to the effect on the nigrostriatal dopaminergic system was dyskinesia. Other non-dopaminergic related adverse events included nausea, headache, back pain, dizziness, and insomnia. Significant adverse events were seen in patients receiving higher doses (600 mg) of levodopa such as nausea, headache, dyskinesia, hypertonia, and infection [63].

#### 3.3.2. Dopamine Agonists

Dopamine agonists can induce more severe side effects as compared to levodopa since they act on peripheral dopamine receptors. Side effects include nausea, orthostatic hypotension, and edema. PD patients may have different reactions to various dopamine agonists with different profiles in terms of side effects, hence changing from one dopamine agonist to the other can be the strategy in overcoming the side effects. Other neuropsychiatric adverse effects include impulse control disorder, dopamimetic-induced psychosis, and dopaminergic dysregulation syndrome. The main adverse effect associated with ergot dopamine agonists is pulmonary fibrosis while the main adverse effect for non-ergot dopamine agonists is excessive daytime sleepiness. Due to the more severe adverse effect of the ergot dopamine agonists, they should only be used as secondary treatment if the non-ergot dopamine agonists are not tolerable [61].

#### 3.3.3. Anticholinergics

Clinical studies showed that when anticholinergics are compared with other antiparkinsonian drugs, they showed a more pronounced adverse effect. Some adverse effects will lead to the discontinuation of the medication. Due to the peripheral anti-muscarinic action, they may cause blurred vision, tachycardia, urinary retention as well as nausea and vomiting [35]. The adverse effects such as difficulty in memorizing, hallucinations, and confusion may be side effects that are bothersome to older adults [100].

#### 3.3.4. COMT Inhibitors

In general, COMT inhibitors were well-tolerated, and no major side effects were identified. The common side effects were dyskinesia, psychiatric effects, hypotension, and nausea due to dopaminergic stimulation. Dopaminergic side effects like dyskinesia often improved after adjustment of the levodopa dosage. Harmless brownish-orange urine discoloration usually can be observed in patients who were taking COMT inhibitors [81]. Tolcapone was correlated in clinical trials with temporary, asymptomatic elevations of transaminases (aspartate transaminase (AST) and alanine transaminase (ALT)) in 1 to 3% of drug-exposed subjects. Three identified hepatotoxicity deaths in tolcapone treated patients prompted its withdrawal from the Canadian and European markets [101].

#### 3.3.5. MOA-B Inhibitors

Based on the DATATOP trial, there was no difference in terms of adverse events, mortality or treatment discontinuance in selegiline as compared to placebo. Besides that, it is discovered that the patients taking selegiline as an adjuvant to levodopa had an increase mortality rate of 60 percent. Tyramine-induced hypertension is the toxicity of MAO-BI as the selectivity to MAO-A will increase at high MAO-BI dose. Besides having a good side effect profile, rasagiline was deemed well-tolerated. Based on the trials, it increased the incidence of depression, hallucinations, and postural hypotension in patients above the age of 70 [39].

#### 3.3.6. Amantadine

In vitro studies showed non-mutagenicity of amantadine while in-vivo studies showed detrimental effects on the nerve central of mice [102]. Amantadine dose up to 15 mg/kg was said to be well-tolerated, nonetheless, dose beyond 15 mg/kg has a negative impact on learning and memory; induces stereotypy; and neurotoxicity. The most reported adverse events with amantadine in the recent meta-analysis were nausea, constipation, edema, hallucination, QT prolongation, and orthostatic hypotension [73].

#### 3.3.7. Antipsychotics Used for Treating PDP

##### Clozapine

Neutropenia and fatal agranulocytosis were reported in most of the clinical trials studies [42,48,49,78,80,81]. However, the risk of neutropenia was small as only a few PD patients in the clozapine group were reported to have neutropenia [76,77]. The risk of agranulocytosis persistent in the first four months of clozapine, for a duration of four weeks. Clozapine can thus be considered safe to be used in PD patients with weekly monitoring of white cell count for 18 weeks and followed by at least every two weeks until the blood count becomes stable [76]. If leukopenia is developed, the condition can be resolved when the patient stops taking the medication [48]. Besides, myocarditis and cardiomyopathy risk were high during the first 2 months [48,49]. Other concerns included anticholinergic effects, metabolic disturbance [42]. Furthermore, a relevant case report showing clozapine in an elderly woman with PDP may induce fatal neuroleptic malignant syndrome [23,103]. In one placebo-controlled double-blind study, a high dose of clozapine with a mean dose of 137.5 mg per day induced delirium and somnolence [48].

##### Olanzapine

The most common adverse events reported for olanzapine therapy were dizziness, somnolence, dry mouth, headache, insomnia, akathisia, and weight gain. Some studies have shown that olanzapine can potentially cause motor deterioration as the extrapyramidal adverse effect even though it is given in low conventional doses [104]. Neuroleptic malignant syndrome may also occur in patients with parkinsonian psychosis as they receive olanzapine as treatment [105].

##### Quetiapine

Quetiapine is well-tolerated; however, the demand for quetiapine decreased due to its low efficacy [42]. The most common side effects of quetiapine were sedation and postural hypotension [42,80]. Based on the findings, FDA has determined that the use of atypical antipsychotics in elderly patients with dementia will increase the risk of mortality, and this risk appears to carry into the PD population [80]. One finding has also shown that there is a higher risk of mortality in patients receiving quetiapine alone rather than receiving pimavanserin alone or in combination [1].

##### Risperidone

Many studies reported that risperidone can worsen motor performance in PD patients. Moreover, there is an improvement in psychotic symptoms in 40% of patients who received risperidone and olanzapine. These two drugs cause the motor symptoms to aggravate and therefore clinicians should avoid prescribing them [42]. As compared to low doses of clozapine and quetiapine, motor function worsening was not seen as that found in the therapy with risperidone and olanzapine [78]. One study showed higher risks of mortality in PD patients treated with risperidone compared to quetiapine [42]. Thus, risperidone should be used with caution while treating PD patients.

##### Ziprasidone

According to Pintor et al., in a 2012 randomized, single-blind, open-label, parallel study, the adverse effects profile of ziprasidone was quite similar to clozapine. The most common adverse effect of ziprasidone (2 patients, 33%) which was somnolence on the first day was less prevalent as compared to clozapine (6 patients, 75%) [52].

Based on Gómez-Esteban et al., 2005, the low prevalence of orthostatic hypotension in ziprasidone as compared to other atypical antipsychotics was attributed to its weak blocking effects towards alpha 1 adrenergic receptor. Furthermore, it did not affect the homeostasis of glucose-insulin and metabolism of lipid [87].

#### 3.3.8. Safinamide

In both Study 016 and SETTLE study, the adverse events (AEs) reported were similar in both safinamide and placebo group. The AEs were mild and moderate. It was similar in both groups for the studies in terms of treatment-emergent adverse events (TEAEs), discontinuations due to TEAEs, drug-related adverse events, and serious adverse events (SAEs). In the safinamide group, the adverse event that was most frequently reported was dyskinesia, but it was mild and moderate and did not lead to the discontinuations. For clinical laboratory tests and vital signs, there were no significant findings [88,90,92]. Other common TEAs reported in Study 016 and SETTLE study were back pain and headache [53].

On the other hand, in the 015 study, the most common reported AEs (<10% in each group) were nausea, headache, vomiting, dizziness, backache, gastritis, and stomach pain [95]. In the 017 study, at least one adverse event which was mild and moderate was reported by the patients [96]. Furthermore, the common AEs (≥5%) reported in the MOTION study were nausea, headache, dizziness, backache, and joint pain [97]. Generally, safinamide is considered to be safe for the add-on treatment of PD and it has a good safety profile.

#### 3.3.9. Istradefylline

Istradefylline, which antagonizes the adenosine A2A receptor selectively, has fewer actions on dopaminergic receptors and dopamine metabolizing enzymes [3,57]. These receptors are found abundantly in basal ganglia [3,106]. Adenosine A2A receptors have a prominent role in the development of dopamine dysregulation syndrome (DDS) [3,107]. The receptors are also responsible for the modulation of reinforcement and reward pathways. Hence, it can lead to the development of impulse control behavior (ICB) by causing excitatory action in the mesolimbic area [3,108]. ICB consists of impulse control disorder such as binge eating, pathological gambling and compulsive buying. The other behaviors include hobbyism, walkabout, punding, and DDS [3].

#### 3.3.10. Pimavanserin

Although pimavanserin was generally well-tolerated by many patients in several studies, some adverse reactions were reported. The most common adverse reactions reported for pimavanserin included confusional state, peripheral edema, and hallucinations [9,86,99]. Furthermore, QT-interval prolongation can be induced by pimavanserin and it may or may not cause serious cardiac outcomes [9,99]. Thus, it is not recommended for patients with pre-existing irregular heart rhythms or QT prolongation. Other problems include falls, instability, confusion, and edema [9,86,99]. Pimavanserin’s safety is unknown in pregnant and lactating women [9]. Moreover, some studies reported that pimavanserin may increase the risk of mortality in patients. Meanwhile, some studies claimed that increase mortality in pimavanserin is due to individuals requiring these medications having greater disease severity and are at higher risk of complications and death [86].

## 4. Overall Comparison of Efficacy and Safety of Conventional Drugs Used in Treating PD

Levodopa, usually in the combination with carbidopa was the gold standard and most effective symptomatic treatment for PD. Levodopa was suggested in all stages of PD including monotherapy, patients with and without motor fluctuations with the presence or absence of dyskinesia, patients with motor complications, and non-motor symptoms. Dopamine agonists were recommended to be used as a single-drug therapy in early PD patients or as an adjuvant to levodopa. Studies have shown that dopamine agonists resulted in a lower incidence of dyskinesia in the initial treatment, however, their benefits need to be proven in long-term studies. On the other hand, anticholinergics were not efficacious in controlling the motor complications of PD as compared to levodopa or dopamine agonists. COMT inhibitors and MAO-B inhibitors were usually used as the adjunct therapy to levodopa or dopamine agonists in reducing motor complications such as dyskinesia and their use has resulted in the reduction of the dose of levodopa when used in combination. Amantadine showed benefit and efficacy in reducing off time and managing levodopa-induced dyskinesia. In overall, levodopa-carbidopa was the mainstay of treatment for PD, and it was found to be superior to other medications such as dopamine agonists, anticholinergics, MAO-B inhibitors, and amantadine. However, in the late disease stage of PD, other adjunct therapies will be introduced to control the motor complications more effectively.

In terms of safety, the most common adverse effects associated with levodopa were mild and moderate such as nausea, headache, and back pain. After the initial treatment period, levodopa will become less effective in reducing the off time and may induce dyskinesia or wearing-off. Dopamine agonists have more serious side effects as compared to levodopa due to their action on peripheral dopamine receptors. The most common dopaminergic adverse effects associated with all dopamine agonists were nausea, orthostatic hypertension, and edema. Impulse control disorder and psychosis were also found in patients on dopamine agonists therapy. Anticholinergics, on the other hand, have more side effects due to their peripheral antimuscarinic effects such as nausea, vomiting, blurred vision, and urinary retention. COMT inhibitors dopaminergic side effects include dyskinesia, hypotension, and nausea. Harmless urine discoloration may also occur while hepatotoxicity has also been reported in patients taking one of the COMT inhibitors named tolcapone. MAO-B inhibitors use in high doses can result in tyramine-induced hypertension. The common adverse effects reported with amantadine were nausea, constipation, edema, and hallucination. Generally, all drugs were well-tolerated with good safety profiles. Levodopa was considered the safest drug used in the treatment of PD as shown by the studies.

## 5. Overall Comparison of New Drugs Approved from 2016–2019 for the Treatment of PD in Terms of Efficacy and Safety

The primary outcome measure for the efficacy of safinamide as found in this systematic review was the change in daily on time with no or non-troublesome dyskinesia. Among the new medications approved from 2016–2019 in treating PD, safinamide was considered as the most effective drug. As shown by the findings in Study 016, both safinamide 50 mg and 100 mg have improved the UPDRS part III score as compared to the placebo. Improvement in CGI-C, CGI-S, and off time was also reported in both groups of patients treated with safinamide 50 mg and 100 mg following the morning levodopa dose. This result was consistent with the findings of the extension study of Study 016, which was Study 018. Safinamide was found to improve the symptoms of tremor, bradykinesia, rigidity, and gait. As compared to safinamide, istradefylline has almost similar efficacy in improving motor performances in PD patients. As shown in one randomized, double-blind, placebo-controlled study, istradefylline 40 mg daily was shown to reduce the UPDRS part III score more prominently compared to istradefylline 20 mg daily. Another study of istradefylline showed that istradefylline 40 mg daily reduced the off time in PD patients more prominently as compared to istradefylline 20 mg daily. Istradefylline was also found to improve the motor fluctuations in PD patients. The new drug used in treating PDP, pimavanserin has also demonstrated efficacy in treating delusions and hallucinations associated with PDP. More improvement in the points for the SAPS-PD score was seen in the group of patients receiving pimavanserin 34 mg as compared to the placebo. Overall, safinamide is effective as the adjuvant therapy to levodopa in increasing on time with non-troublesome or no dyskinesia.

In terms of safety, safinamide was known to have relatively good safety profiles and was well-tolerated. The most common adverse event reported with safinamide was dyskinesia, however, this side effect was mild and moderate and did not lead to discontinuations among patients most of the time. Other common adverse events reported included nausea, headache, vomiting, dizziness, backache, and stomach pain. Istradefylline was reported to cause impulse control behavior and dopamine dysregulation syndrome. On the other hand, the common adverse events reported with pimavanserin were hallucinations, confusional state, peripheral edema, and QT-interval prolongation. In short, safinamide was considered as the safest drug as compared to istradefylline and pimavanserin due to its mild adverse effects. The summary of safinamide, istradefylline, and pimavanserin in terms of efficacy and safety are shown in Table 1.

In comparison to older drugs approved for the treatment of PD, the newer drugs approved between 2016 and 2019 have improved safety and efficacy profiles. Among all the newer drugs, safinamide was found to have relatively good efficacy and safety profiles in controlling PD symptoms.

## 6. Non-Pharmacological Management

Besides pharmacological management, non-pharmacological management is also an essential part and is considered as an adjunct to drug therapy in treating PD patients. Some of the non-pharmacological approaches include physiotherapy, speech and language therapy, occupational therapy, and cognitive training. Physiotherapy generally involves exercise for better physical functioning, strength training for the reduction of motor symptoms as well as balance and gait training. Balance and gait training have been shown by a study that they improve performance in PD patients, and this is associated with the adaptive plasticity of their brains after training. In order to increase quality of life and help PD patients to have more social participation, occupational therapy is also important. Moreover, swallowing therapy and speech therapy can improve patients’ quality of life as most of the PD patients have dysphagia problems. For other non-motor symptoms such as anxiety and depression, cognitive training can offer some benefits and the effects are believed to be mediated by the increase in neurotransmitter modulation and levels of brain-derived neurotrophic factor [110]. Some studies have shown that electroconvulsive therapy can improve the motor symptoms of PD patients, but its effectiveness in treating PD psychosis needs to be confirmed by further studies [48]. For the treatment of PD psychosis, psychotherapeutic interventions and educational approaches can be the adjunct therapy. In addition to the PD therapy, deep brain stimulation (DBS) also helps the PD individual to improve their conditions. This is a surgical procedure in which electrodes are implanted in specific brain areas. These electrodes, or leads, generate electrical impulses that control abnormal brain activity. Electrical impulses can also compensate for chemical imbalances within the brain that cause a variety of conditions. A programmable generator placed under the skin in the upper chest controls brain stimulation [111].

## 7. Conclusions

In summary, this review provides an introduction to the efficacy and safety of all old drugs and new drugs approved from 2016–2019 in treating PD. Based on the comparison of findings from all trials and studies, it was found that safinamide demonstrated the highest efficacy and safety as add-on therapy in controlling PD symptoms. The most common adverse event associated with safinamide was dyskinesia and gastrointestinal symptoms, both of which were mild and tolerable. All the other agents also demonstrated favorable efficacy in controlling PD motor complications. However, further studies and trials need to be conducted in order to prove and establish their long-term efficacy and safety profiles.

## Figures and Tables

**Table 1:** Efficacy and safety of the newly approved drugs for treatment of PD.

Drug Name	Author, Year, Reference Number	Study Design	Population Characteristics	Interventions	Primary Outcome Measured	Efficacy	Safety
Safinamide	Schapira A., et al., 2013 [88]	Randomized, placebo-controlled, double-blind international Phase III trial.	Patients who had mid-to-late-stage idiopathic PD (>3 years of disease) and were treated with optimized, stable doses of L-dopa and DA, catechol-O-methyltransferase inhibitor, anticholinergic, and/or amantadine.	Safinamide 50 mg, Safinamide 100 mg, placebo.	Change in daily on time with no or non-troublesome dyskinesia.	Improved on time (without worsening the troublesome dyskinesia), off time, UPDRS part III, CGI-S, CGI-C, PDQ-39 and off time following the first morning L-dopa dose.	Major AEs: Back pain, headache, falls, dyskinesias, nausea, and urinary tract infections
	Borgohain R., et al., 2014 [90]	Randomized, placebo-controlled, double-blind Phase III trial. (Study 016)	Patients aged 30–80 years, had been diagnosed with PD ≥3 years, had the presence of motor fluctuations with 1.5 h off a day.	Safinamide 50 mg, Safinamide 100 mg, placebo.	Change in mean daily on time with no or non-troublesome dyskinesias in the 18-h recording period.	Improved UPDRS part III in both safinamide 50 mg (*p* = 0.0138) and 100 mg (*p* = 0.0006) groups. Significant improvement in off time, CGI-C and CGI-S in both safinamide groups following the morning dose of levodopa.	Major AEs: Back pain, headache, dyskinesia, depression, and hypertension
	Borgohain R., et al., 2014 [91]	Randomized, double-blind, placebo-controlled, 18-month extension study. (Study 018)	Patients who had completed the 016 study or patients who had completed efficacy evaluation at weeks 12 and 24 of Study 016.	Safinamide 50 mg, Safinamide 100 mg, placebo.	Mean change from baseline at Study 016 to endpoint of the DRS score during on time.	Improved total daily on time without troublesome dyskinesia from baseline for safinamide 50 mg (*p* = 0.0031) and safinamide 100 mg (*p* = 0.0002). Improved off time, CGI-S, CGI-C (for SAF 50 mg), UPDRS part II, part III and part IV total scores and PDQ-39.	Major AEs: Back pain, insomnia, headache, and dyskinesia
	Stocchi F., et al., 2004 [93]	Randomized, placebo-controlled, double-blind, Phase II, dose finding study.	Early PD patients.	Safinamide 0.5 mg/kg, Safinamide 1.0 mg/kg, placebo as monotherapy or as adjunct therapy to a single DA.	Proportion of patients considered as treatment responders, for example 30% improvement in UPDRS part III compared with baseline.	Improved UPDRS part III as compared to baseline, more statistically significant between safinamide 1.0 mg/kg and placebo (*p* = 0.016).	Major AEs: Abdominal pain, dizziness, and musculoskeletal and connective tissue disorders
	Stocchi F., et al., 2006 [94]	Single-center, open, pilot trial.	25 PD patients with Hoehn and Yahr (H&Y) stages III–IV.	Safinamide 100 mg, Safinamide 150 mg, Safinamide 200 mg as adjunct therapy to stable single DA or LD.	Changes in UPDRS part II, part III, and part IV and CGI.	Improved motor performance (evaluated by UPDRS part III) for more than an 8-week period (*p* < 0.001).	Major AEs: -
	Stocchi F., et al., 2012 [95]	Randomized, placebo-controlled, double-blind Phase III trial. (Study 015)	Early PD patients aged 30–80 years, who were diagnosed with idiopathic PD with <5 years of history and had Hoehn and Yahr (H&Y) stages I–II.	Safinamide 100 mg, Safinamide 200 mg, placebo as adjunct therapy to stable single DA.	Changes in UPDRS part III total score from baseline to endpoint (week 24).	Improved UPDRS part II, UPDRS part III and CGI-C total score in safinamide 100 mg group (*p* = 0.0419).	Major AEs: Nausea, vomiting, headache, dizziness, back pain, gastritis, and abdominal pain
	Schapira A., et al., 2013. [96]	Randomized, double-blind, placebo-controlled, 12-month extension study. (Study 017)	Patients who had completed Study 015 or patients who had completed efficacy evaluation at weeks 12 and 24 of Study 015.	Safinamide 100 mg, Safinamide 200 mg, placebo as adjunct therapy to stable single DA.	Time to intervention from baseline.	Lower rate of intervention in the safinamide 100 mg group compared with dopamine agonists monotherapy (*p* < 0.05). Improved UPDRS part II and part III was greater in safinamide 100 mg group.	Major AEs: Dizziness, nausea, back pain, nausea, and upper abdominal pain
	Barone P., et al., 2013 [97]	Randomized, placebo-controlled, double-blind international Phase III trial.	Patients with early idiopathic PD (<5 years) who were treated with a single DA.	Safinamide 50 mg, Safinamide 100 mg, placebo.	Change in UPDRS part III from baseline to week 24.	Improved UPDRS part III (*p* = 0.0396) and PDQ-39 in the safinamide 100 mg group.	Major AEs: Nausea, dizziness, headache, arthralgia, and back pain
Istradefylline	Mizuno Y., et al., 2013 [57]	Multicenter, placebo-controlled, randomized, double-blind, parallel-group study.	PD patients with motor complication.	Istradefylline 20 or 40 mg/day and placebo.	Change in daily off time.	The change in daily off time was significantly reduced in the istradefylline 20 mg/day (−0.99 h, *p* = 0.003) and istradefylline 40 mg/day (−0.96 h, *p* = 0.003) groups.	Major AEs: Dyskinesia, gait disturbance, gastric ulcer, and hallucinations
Pimavanserin	Espay A., et al., 2018 [99]	6-week randomized, double-blind, placebo-controlled, phase 3 trial.	Patients with PD psychosis.	Pimavanserin 34 mg and placebo.	Change in the Scale for the Assessment of Positive Symptoms-PD.	Mean (pimavanserin vs. placebo) change from baseline was larger in the cognitively impaired (*n* = 50; −6.62 vs. −0.91; *p* *=* 0.002) versus the cognitively unimpaired (*n* = 135; −5.50 vs. −3.23; *p* *=* 0.046) group. The mean difference in SAPS-PD score change from baseline for pimavanserin versus placebo was −3.06 at day 43 (*p* *=* 0.001).	Major AEs: Urinary tract infection, fall, peripheral edema, hallucinations, nausea, and confusional state
	Cummings J., et al., 2018 [109]	6-week, randomized, double-blind, placebo-controlled study.	Patients with PD psychosis.	Pimavanserin 40 mg and placebo.	SAPS-PD score change from baseline to week 6.	Pimavanserin was associated with statistically significant 5.79 point improvement at week 6 as compared to placebo with 2.73 point (*p* = 0.001)	Major AEs: Nausea, headache, fall, urinary tract infection, peripheral edema, confusional state, and hallucinations

## Data Availability

The data presented in this study are available on request from the corresponding author.

## References

[1] Moreno G.M., Gandhi R., Lessig S.L., Wright B., Litvan I., Nahab F.B. (2018). Mortality in patients with Parkinson disease psychosis receiving pimavanserin and quetiapine. Neurology.

[2] Qureshi A.R., Rana A.Q., Malik S.H., Rizvi S.F.H., Akhter S., Vannabouathong C., Sarfraz Z., Rana R. (2018). Comprehensive Examination of Therapies for Pain in Parkinson’s Disease: A Systematic Review and Meta-Analysis. Neuroepidemiology.

[3] Kon T., Ueno T., Haga R., Tomiyama M. (2018). The factors associated with impulse control behaviors in Parkinson’s disease: A 2-year longitudinal retrospective cohort study. Brain Behav..

[4] Binde C.D., Tvete I.F., Gåsemyr J., Natvig B., Klemp M. (2018). A multiple treatment comparison meta-analysis of monoamine oxidase type B inhibitors for Parkinson’s disease. Br. J. Clin. Pharmacol..

[5] Müller T., Riederer P., Grünblatt E. (2017). Determination of Monoamine Oxidase A and B Activity in Long-Term Treated Patients With Parkinson Disease. Clin. Neuropharmacol..

[6] Sadek B., Saad A., Schwed J.S., Weizel L., Walter M., Stark H. (2016). Anticonvulsant effects of isomeric nonimidazole histamine H_3_ receptor antagonists. Drug Des. Dev. Ther..

[7] Cattaneo C., Barone P., Bonizzoni E., Sardina M. (2017). Effects of Safinamide on Pain in Fluctuating Parkinson’s Disease Patients: A Post-Hoc Analysis. J. Park. Dis..

[8] Marras C., Beck J.C., Bower J.H., Roberts E., Ritz B., Ross G.W., Abbott R.D., Savica R., Eeden S.K.V.D., Willis A.W. (2018). Prevalence of Parkinson’s disease across North America. Jpn. Park. Dis..

[9] Mohanty D., Sarai S., Naik S., Lippmann S. (2019). Pimavanserin for Parkinson Disease Psychosis. Prim. Care Companion CNS Disord..

[10] Hammarlund C.S., Westergren A., Åström I., Edberg A.-K., Hagell P. (2018). The Impact of Living with Parkinson’s Disease: Balancing within a Web of Needs and Demands. Park. Dis..

[11] Fackrell R., Carroll C.B., Grosset D.G., Mohamed B., Reddy P., Parry M., Chaudhuri K.R., Foltynie T. (2018). Noninvasive options for ‘wearing-off’ in Parkinson’s disease: A clinical consensus from a panel of UK Parkinson’s disease specialists. Neurodegener. Dis. Manag..

[12] Tysnes O.-B., Storstein A. (2017). Epidemiology of Parkinson’s disease. J. Neural Transm..

[13] Fredericks D., Norton J.C., Atchison C., Schoenhaus R., Pill M.W. (2017). Parkinson’s disease and Parkinson’s disease psychosis: A perspective on the challenges, treatments, and economic burden. Am. J. Manag. Care.

[14] Dorsey E.R., Elbaz A., Nichols E., Abbasi N., Abd-Allah F., Abdelalim A., Adsuar J.C., Ansha M.G., Brayne C., Choi J.-Y.J. (2018). Global, regional, and national burden of Parkinson’s disease, 1990–2016: A systematic analysis for the Global Burden of Disease Study 2016. Lancet Neurol..

[15] (2018). Department of Statistics Malaysia. https://www.dosm.gov.my/v1/.

[16] Papapetropoulos S., Adi N., Ellul J., Argyriou A.A., Chroni E. (2007). A Prospective Study of Familial versus Sporadic Parkinson’s Disease. Neurodegener. Dis..

[17] Causes of Parkinson’s Disease. The Michael J. Fox Foundation for Parkinson’s Research|Parkinson’s Disease. https://www.michaeljfox.org/causes.

[18] Johnson M.E., Stecher B., Labrie V., Brundin L., Brundin P. (2019). Triggers, Facilitators, and Aggravators: Redefining Parkinson’s Disease Pathogenesis. Trends Neurosci..

[19] Shin H.-W., Chung S.J. (2012). Drug-Induced Parkinsonism. J. Clin. Neurol..

[20] Association EPD Vascular (Arteriosclerotic) Parkinsonism. https://www.epda.eu.com/about-parkinsons/types/vascular-arteriosclerotic-parkinsonism/.

[21] Galvan A., Wichmann T. (2008). Pathophysiology of Parkinsonism. Clin. Neurophysiol. Off. J. Int. Fed. Clin. Neurophysiol..

[22] Mhyre T.R., Boyd J.T., Hamill R.W., Maguire-Zeiss K.A. (2012). Parkinson’s Disease. Subcell. Biochem..

[23] Bamford N.S., Robinson S., Palmiter R.D., Joyce J., Moore C., Meshul C.K. (2004). Dopamine Modulates Release from Corticostriatal Terminals. J. Neurosci..

[24] Etiology and Pathogenesis of Parkinson Disease—UpToDate. https://www.uptodate.com/contents/etiology-and-pathogenesis-of-parkinson-disease.

[25] Kenneth L., Zigmoid M.J., Burke R.E. (2002). Neuropsychopharmacology: The Fifth Generation of Progress.

[26] Obeso J.A., Oroz M.C.R., Rodriguez M., Lanciego J., Artieda J., Gonzalo N., Olanow C.W. (2000). Pathophysiology of the basal ganglia in Parkinson’s disease. Trends Neurosci..

[27] Bezard E., Gross C.E., Brotchie J. (2003). Presymptomatic compensation in Parkinson’s disease is not dopamine-mediated. Trends Neurosci..

[28] Adams J.R., van Netten H., Schulzer M., Mak E., Mckenzie J., Strongosky A., Sossi V., Ruth T.J., Lee C.S., Farrer M. (2005). PET in LRRK2 mutations: Comparison to sporadic Parkinson’s disease and evidence for presymptomatic compensation. Brain J. Neurol..

[29] Hashimoto M., Rockenstein E., Crews L., Masliah E. (2003). Role of Protein Aggregation in Mitochondrial Dysfunction and Neurodegeneration in Alzheimer’s and Parkinson’s Diseases. NeuroMolecular Med..

[30] Antonini A. (2010). Levodopa in the treatment of Parkinson’s disease: An old drug still going strong. Clin. Interv. Aging.

[31] Hauser R.A. (2009). Levodopa: Past, Present, and Future. Eur. Neurol..

[32] Sgroi S., Kaelin-Lang A., Capper-Loup C. (2014). Spontaneous locomotor activity and L-DOPA-induced dyskinesia are not linked in 6-OHDA parkinsonian rats. Front. Behav. Neurosci..

[33] Brooks D. (2000). Dopamine agonists: Their role in the treatment of Parkinson’s disease. J. Neurol. Neurosurg. Psychiatry.

[34] Kelley B.J., Duker A., Chiu P. (2012). Dopamine Agonists and Pathologic Behaviors. Park. Dis..

[35] Katzenschlager R., Sampaio C., Costa J., Lees A. (2002). Anticholinergics for symptomatic management of Parkinson’s disease. Cochrane Database Syst. Rev..

[36] Dong J., Cui Y., Li S., Le W. (2016). Current Pharmaceutical Treatments and Alternative Therapies of Parkinson’s Disease. Curr. Neuropharmacol..

[37] Rivest J., Barclay C.L., Suchowersky O. (1999). COMT Inhibitors in Parkinson’s Disease. Can. J. Neurol. Sci..

[38] Tripathi R.K.P., Ayyannan S.R. (2019). Monoamine oxidase-B inhibitors as potential neurotherapeutic agents: An overview and update. Med. Res. Rev..

[39] Dezsi L., Vecsei L. (2017). Monoamine Oxidase B Inhibitors in Parkinson’s Disease. CNS Neurol. Disord.—Drug Targets.

[40] Chang C., Ramphul K. (2020). Amantadine. https://www.ncbi.nlm.nih.gov/books/NBK499953/.

[41] Crosby N.J., Deane K., Clarke C. (2003). Amantadine for dyskinesia in Parkinson’s disease. Cochrane Database Syst. Rev..

[42] Divac N., Stojanović R., Vujović K.S., Medić B., Damjanović A., Prostran M. (2016). The Efficacy and Safety of Antipsychotic Medications in the Treatment of Psychosis in Patients with Parkinson’s Disease. Behav. Neurol..

[43] Stępnicki P., Kondej M., Kaczor A.A. (2018). Current Concepts and Treatments of Schizophrenia. Molecules.

[44] (2020). Accessdata.fda.gov. https://www.accessdata.fda.gov/drugsatfda_docs/label/2010/019758s062lbl.pdf.

[45] Ballanger B., Strafella A.P., van Eimeren T., Zurowski M., Rusjan P.M., Houle S., Fox S.H. (2010). Serotonin 2A Receptors and Visual Hallucinations in Parkinson Disease. Arch. Neurol..

[46] Thomas K., Saadabadi A. (2020). Olanzapine. StatPearls.

[47] Tollens F., Gass N., Becker R., Schwarz A., Risterucci C., Künnecke B., Lebhardt P., Reinwald J., Sack M., Weber-Fahr W. (2018). The affinity of antipsychotic drugs to dopamine and serotonin 5-HT2 receptors determines their effects on prefrontal-striatal functional connectivity. Eur. Neuropsychopharmacol..

[48] Martinez-Ramirez D., Okun M.S., Jaffee M.S. (2016). Parkinson’s disease psychosis: Therapy tips and the importance of communication between neurologists and psychiatrists. Neurodegener. Dis. Manag..

[49] Yuan M., Sperry L., Malhado-Chang N., Duffy A., Wheelock V., Farias S., O’Connor K., Olichney J., Shahlaie K., Zhang L. (2017). Atypical antipsychotic therapy in Parkinson’s disease psychosis: A retrospective study. Brain Behav..

[50] Torti M., Vacca L., Stocchi F. (2018). Istradefylline for the treatment of Parkinson’s disease: Is it a promising strategy?. Expert Opin. Pharmacother..

[51] Ellis T., Cudkowicz M.E., Sexton P.M., Growdon J.H. (2000). Clozapine and Risperidone Treatment of Psychosis in Parkinson’s Disease. J. Neuropsychiatry Clin. Neurosci..

[52] Pintor L., Valldeoriola F., Bailles E., Martí M.J., Muñiz A., Tolosa E. (2012). Ziprasidone Versus Clozapine in the Treatment of Psychotic Symptoms in Parkinson Disease. Clin. Neuropharmacol..

[53] Stocchi F., Torti M. (2016). Adjuvant therapies for Parkinson’s disease: Critical evaluation of safinamide. Drug Des. Dev. Ther..

[54] Cattaneo C., Kulisevsky J., Tubazio V., Castellani P. (2018). Long-term Efficacy of Safinamide on Parkinson’s Disease Chronic Pain. Adv. Ther..

[55] Agnati L.F., Ferre S., Lluis C., Franco R., Fuxe K. (2003). Molecular Mechanisms and Therapeutical Implications of Intramembrane Receptor/Receptor Interactions among Heptahelical Receptors with Examples from the Striatopallidal GABA Neurons. Pharmacol. Rev..

[56] Jenner P. (2005). Istradefylline, a novel adenosine A2Areceptor antagonist, for the treatment of Parkinson’s disease. Expert Opin. Investig. Drugs.

[57] Mizuno Y., Kondo T., The Japanese Istradefylline Study Group (2013). Adenosine A2Areceptor antagonist istradefylline reduces daily OFF time in Parkinson’s disease. Mov. Disord..

[58] Cummings J., Ballard C., Tariot P., Owen R., Foff E., Youakim J., Norton J., Stankovic S. (2018). Pimavanserin: Potential Treatment for Dementia-Related Psychosis. J. Prev. Alzheimer’s Dis..

[59] Vanover K.E., Weiner D.M., Makhay M., Veinbergs I., Gardell L.R., Lameh J., Del Tredici A.L., Piu F., Schiffer H.H., Ott T.R. (2006). Pharmacological and Behavioral Profile of N-(4-Fluorophenylmethyl)-N-(1-methylpiperidin-4-yl)-N′-(4-(2-methylpropyloxy)phenylmethyl) Carbamide (2R,3R)-Dihydroxybutanedioate (2:1) (ACP-103), a Novel 5-Hydroxytryptamine2A Receptor Inverse Agonist. J. Pharmacol. Exp. Ther..

[60] Jalal B. (2018). The neuropharmacology of sleep paralysis hallucinations: Serotonin 2A activation and a novel therapeutic drug. Psychopharmacology.

[61] Oertel W., Schulz J.B. (2016). Current and experimental treatments of Parkinson disease: A guide for neuroscientists. J. Neurochem..

[62] PD MED Collaborative Group incl (2014). Long-term effectiveness of dopamine agonists and monoamine oxidase B inhibitors compared with levodopa as initial treatment for Parkinson’s disease (PD MED): A large, open-label, pragmatic randomised trial. Lancet.

[63] Fahn S., Oakes D., Shoulson I., Kieburtz K., Rudolph A., Lang A., Olanow C.W., Tanner C., Marek K., Parkinson Study Group (2004). Levodopa and the Progression of Parkinson’s Disease. N. Engl. J. Med..

[64] Fox S.H., Katzenschlager R., Lim S.-Y., Ravina B., Seppi K., Coelho M., Poewe W., Rascol O., Goetz C.G., Sampaio C. (2011). The Movement Disorder Society Evidence-Based Medicine Review Update: Treatments for the motor symptoms of Parkinson’s disease. Mov. Disord..

[65] Parkinson Study Group CALM Cohort Investigators (2009). Long-term Effect of Initiating Pramipexole vs. Levodopa in Early Parkinson Disease. Arch. Neurol..

[66] Rascol O., Brooks D., Korczyn A., De Deyn P.P., Clarke C.E., Lang A. (2000). A Five-Year Study of the Incidence of Dyskinesia in Patients with Early Parkinson’s Disease Who Were Treated with Ropinirole or Levodopa. N. Engl. J. Med..

[67] Oertel W.H., Wolters E., Sampaio C., Gimenez-Roldan S., Bergamasco B., Dujardin M., Grosset D., Arnold G., Leenders K.L., Hundemer H.-P. (2005). Pergolide versus levodopa monotherapy in early Parkinson’s disease patients: The PELMOPET study. Mov. Disord..

[68] Lang A.E., Lees A. (2002). Management of Parkinson’s disease: An evidence-based review. Mov. Disord..

[69] Katsaiti I., Nixon J. (2018). Are There Benefits in Adding Catechol-O Methyltransferase Inhibitors in the Pharmacotherapy of Parkinson’s Disease Patients? A Systematic Review. J. Park. Dis..

[70] Tolcapone (2020). Ncbi.nlm.nih.gov. https://www.ncbi.nlm.nih.gov/books/NBK548573/.

[71] Rocha J.F., Almeida L., Falcão A., Palma P.N., Loureiro A.I., Pinto R., Bonifácio M.J., Wright L.C., Nunes T., Soares-Da-Silva P. (2013). Opicapone: A short lived and very long acting novel catechol-O-methyltransferase inhibitor following multiple dose administration in healthy subjects. Br. J. Clin. Pharmacol..

[72] Ferreira J.J., Lees A., Rocha J.-F., Poewe W., Rascol O., Soares-Da-Silva P. (2016). Opicapone as an adjunct to levodopa in patients with Parkinson’s disease and end-of-dose motor fluctuations: A randomised, double-blind, controlled trial. Lancet Neurol..

[73] Perez-Lloret S., Rascol O. (2018). Efficacy and safety of amantadine for the treatment of l-DOPA-induced dyskinesia. J. Neural Transm..

[74] Pajo A.T., I Espiritu A., Jamora R.D.G. (2019). Efficacy and safety of extended-release amantadine in levodopa-induced dyskinesias: A meta-analysis. Neurodegener. Dis. Manag..

[75] Oertel W., Eggert K., Pahwa R., Tanner C.M., Hauser R.A., Trenkwalder C., Ehret R., Azulay J.P., Isaacson S., Felt L. (2017). Randomized, placebo-controlled trial of ADS-5102 (amantadine) extended-release capsules for levodopa-induced dyskinesia in Parkinson’s disease (EASE LID 3). Mov. Disord..

[76] Parkinson Study Group (1999). Low-Dose Clozapine for the Treatment of Drug-Induced Psychosis in Parkinson’s Disease. N. Engl. J. Med..

[77] Pollak P., Tison F., Rascol O., Destée A., Péré J.J., Senard J.-M., Durif F., Bourdeix I. (2004). Clozapine in drug induced psychosis in Parkinson’s disease: A randomised, placebo controlled study with open follow up. J. Neurol. Neurosurg. Psychiatry.

[78] Hermanowicz N., Alva G., Pagan F., Patel A., Madrid K.C., Kremens D., Kenney J., Arquette S., Tereso G., Farnum C. (2017). The Emerging Role of Pimavanserin in the Management of Parkinson’s Disease Psychosis. J. Manag. Care Spec. Pharm..

[79] Ffytche D.H., Creese B., Politis M., Chaudhuri K.R., Weintraub D., Ballard C., Aarsland D. (2017). The psychosis spectrum in Parkinson disease. Nat. Rev. Neurol..

[80] Samudra N., Patel N., Womack K., Khemani P., Chitnis S. (2016). Psychosis in Parkinson Disease: A Review of Etiology, Phenomenology, and Management. Drugs Aging.

[81] (2020). UpToDate. Uptodate.com. https://www.uptodate.com/contents/medical-management-of-motor-fluctuations-and-dyskinesia-in-parkinson-disease#.

[82] Frieling H., Hillemacher T., Ziegenbein M., Neundörfer B., Bleich S. (2007). Treating dopamimetic psychosis in Parkinson’s disease: Structured review and meta-analysis. Eur. Neuropsychopharmacol..

[83] (2020). Recommendations|Parkinson’s Disease in Adults Guidance|NICE. https://www.nice.org.uk/guidance/ng71/chapter/Recommendations#pharmacological-management-of-motor-symptoms.

[84] Ondo W.G., Levy J.K., Vuong K.D., Hunter C., Jankovic J. (2002). Olanzapine treatment for dopaminergic-induced hallucinations. Mov. Disord. Off. J. Mov. Disord. Soc..

[85] Goetz C., Blasucci L., Leurgans S., Pappert E. (2000). Olanzapine and clozapine: Comparative effects on motor function in hallucinating PD patients. Neurology.

[86] Sellers J., Darby R.R., Farooque A., Claassen D.O. (2019). Pimavanserin for Psychosis in Parkinson’s Disease-Related Disorders: A Retrospective Chart Review. Drugs Aging.

[87] Gómez-Esteban J.C., Zarranz J.J., Velasco F., Lezcano E., Lachen M.C., Rouco I., Barcena J., Boyero S., Ciordia R., Allue I. (2005). Use of Ziprasidone in Parkinsonian Patients With Psychosis. Clin. Neuropharmacol..

[88] Schapira A., Fox S., Hauser R., Jankovic J., Jost W., Kulisevsky J., Pahwa R., Poewe W., Anand R. Safinamide add on to L-dopa: At randomized, placebo controlled 24 weeks global trial in patients with Parkinson’s disease and motor fluctuations. Proceedings of the 65th Annual Meeting of the American Academy of Neurology (AAN).

[89] Pagonabarraga J., Kulisevsky J. (2017). Safinamide from daily clinical practice: First clinical steps. Rev. Neurol..

[90] Borgohain R., Szasz J., Stanzione P., Meshram C., Bhatt M., Chirilineau D., Stocchi F., Lucini V., Giuliani R., Forrest E. (2014). Randomized trial of safinamide add-on to levodopa in Parkinson’s disease with motor fluctuations. Mov. Disord..

[91] Borgohain R., Szasz J., Stanzione P., Meshram C., Bhatt M.H., Chirilineau D., Stocchi F., Lucini V., Giuliani R., Forrest E. (2014). Two-year, randomized, controlled study of safinamide as add-on to levodopa in mid to late Parkinson’s disease. Mov. Disord..

[92] Cattaneo C., Sardina M., Bonizzoni E. (2016). Safinamide as Add-On Therapy to Levodopa in Mid- to Late-Stage Parkinson’s Disease Fluctuating Patients: Post hoc Analysesof Studies 016 and SETTLE. J. Park. Dis..

[93] Stocchi F., Arnold G., Onofrj M., Kwiecinski H., Szczudlik A., Thomas A., Bonuccelli U., Van Dijk A., Cattaneo C., Sala P. (2004). Improvement of motor function in early Parkinson disease by safinamide. Neurology.

[94] Stocchi F., Vacca L., Grassini P., De Pandis M.F., Battaglia G., Cattaneo C., Fariello R.G. (2006). Symptom relief in Parkinson disease by safinamide: Biochemical and clinical evidence of efficacy beyond MAO-B inhibition. Neurology.

[95] Stocchi F., Dm R.B., Onofrj M., Schapira A.H., Bhatt M., Lucini V., Giuliani R., Anand R., for the Study 015 Investigators (2011). A randomized, double-blind, placebo-controlled trial of safinamide as add-on therapy in early Parkinson’s disease patients. Mov. Disord..

[96] Schapira A.H.V., Stocchi F., Borgohain R., Onofrj M., Bhatt M., Lorenzana P., Lucini V., Giuliani R., Anand R., The Study 017 Investigators (2013). Long-term efficacy and safety of safinamide as add-on therapy in early Parkinson’s disease. Eur. J. Neurol..

[97] Barone P., Fernandez H., Ferreira J., Mueller T., Saint-Hilaire M., Stacy M., Tolosa E., Anand R. Safinamide as an add-on therapy to a stable dose of a single dopamine agonist: Results from a randomized, placebo-controlled, 24-week multicenter trial in early idiopathic Parkinson disease patients (MOTION Study). Proceedings of the 65th Annual Meeting of the American Academy of Neurology (AAN).

[98] De La Cruz J., Canal C. (2019). Can pimavanserin help patients with Parkinson disease psychosis?. J. Am. Acad. Phys. Assist..

[99] Espay A.J., Guskey M.T., Norton J.C., Coate B., Vizcarra J.A., Ballard C., Factor S.A., Friedman J.H., Lang A.E., Larsen N.J. (2018). Pimavanserin for Parkinson’s Disease psychosis: Effects stratified by baseline cognition and use of cognitive-enhancing medications. Mov. Disord..

[100] Patient Education: Parkinson Disease Treatment Options—Medications (Beyond the Basics)—UpToDate. https://www.uptodate.com/contents/parkinson-disease-treatment-options-medications-beyond-the-basics#H16.

[101] Watkins P. (2000). COMT inhibitors and liver toxicity. Neurology.

[102] Kaefer V., Semedo J.G., Kahl V.F.S., Von Borowsky R.G., Gianesini J., Kist T.B.L., Pereira P., Picada J.N. (2010). DNA damage in brain cells and behavioral deficits in mice after treatment with high doses of amantadine. J. Appl. Toxicol..

[103] Mocci G., Jiménez-Sánchez L., Adell A., Cortes R., Artigas F. (2014). Expression of 5-HT2A receptors in prefrontal cortex pyramidal neurons projecting to nucleus accumbens. Potential relevance for atypical antipsychotic action. Neuropharmacology.

[104] Fernandez H.H., Trieschmann M.E., Friedman J.H. (2003). Treatment of psychosis in Parkinson’s disease: Safety considerations. Drug Saf..

[105] Lertxundi U., Ruiz A.I., Aspiazu M.Á.S., Domingo-Echaburu S., García M., Aguirre C., García-Moncó J.C. (2015). Adverse Reactions to Antipsychotics in Parkinson Disease. Clin. Neuropharmacol..

[106] Rosin D.L., Hettinger B.D., Lee A., Linden J. (2003). Anatomy of adenosine A2A receptors in brain: Morphological substrates for integration of striatal function. Neurology.

[107] Evans A.H., Pavese N., Lawrence A.D., Tai Y.F., Appel S., Doder M., Brooks D.J., Lees A.J., Piccini P. (2006). Compulsive drug use linked to sensitized ventral striatal dopamine transmission. Ann. Neurol..

[108] Filip M., Zaniewska M., Frankowska M., Wydra K., Fuxe K. (2012). The Importance of the Adenosine A2A Receptor-Dopamine D2 Receptor Interaction in Drug Addiction. Curr. Med. Chem..

[109] Cummings J., Isaacson S., Mills R., Williams H., Chi-Burris K., Corbett A., Dhall R., Ballard C. (2014). Pimavanserin for patients with Parkinson’s disease psychosis: A randomised, placebo-controlled phase 3 trial. Lancet.

[110] Bloem B.R., De Vries N.M., Ebersbach G. (2015). Nonpharmacological treatments for patients with Parkinson’s disease. Mov. Disord..

[111] Julie G.P., Olga K., Shrey P., American Association of Neurological Surgeons Deep Brain Stimulation. https://www.aans.org/en/Patients/Neurosurgical-Conditions-and-Treatments/Deep-Brain-Stimulation.

